# The End of the Partnership With a Guide Dog: Emotional Responses, Effects on Quality of Life and Relationships With Subsequent Dogs

**DOI:** 10.3389/fvets.2021.543463

**Published:** 2021-04-22

**Authors:** Janice Lloyd, Claire Budge, Steve La Grow, Kevin Stafford

**Affiliations:** ^1^Discipline of Veterinary Sciences, James Cook University, Townsville, QLD, Australia; ^2^College of Health, Massey University, Palmerston North, New Zealand; ^3^Institute of Veterinary, Animal and Biomedical Sciences, Massey University, Palmerston North, New Zealand

**Keywords:** guide dog, human—animal relationships, quality of life, grief, attachment, assistance dog, blind mobility aid, vision impaired

## Abstract

Guide dogs are mobility aids that facilitate independent travel of people who are blind or visually impaired. Additional benefits imparted to the guide dog handler include companionship, and increased: social-function, self-esteem and confidence. Some evidence shows that the end of the guide dog partnership can result in reduced mobility, and may have profound psychosocial effects on the handler due to feelings of bereavement and loss of self-esteem. However, this evidence is limited. This study examined the experiences and feelings of 36 people across New Zealand, who experienced the ending of at least one partnership with a guide dog (77 pairings), to explore issues arising at the end of the partnership and how this may impact on relationships with subsequent dogs. Results indicate that the majority of handlers experienced a reduction in their quality of life due to a decrease in independent mobility followed by the loss of a friend and companion, curtailment of social interactions, and loss of self-esteem/confidence. The end of the partnership affected people in different ways. Most handlers “accepted” the partnership had ended, but some felt guilty or angry with the guide dog school. Most applied for another dog immediately, as the need for mobility was high, while others preferred to wait and a smaller number did not reapply. Feelings at this time also affected the handlers' relationships with subsequent guide dogs, with over a quarter expressing a negative effect. Retiring a guide dog (for whatever reason) is not only difficult for the handler, but also for the handler's family, friends, co-workers, and doubtlessly, the dog. The majority of handlers expressed feelings of extreme grief when the partnership ended, whether it was successful or not. Feelings of extreme grief were more common for first than subsequent dogs. The depth of emotion was compared to losing a family member or other loved one, which has been reported in some person and pet relationships. A better understanding of issues surrounding the end of the partnership, including the human-animal bond, will help inform the guide dog industry of how best to support their clients during this time and when transitioning to another dog. Findings may be applied to other service/assistance dog users and the pet owning community.

## Introduction

Guide dogs are primary mobility aids intended to enhance the lifestyle of people with a visual disability (blind or visually impaired) by facilitating independent travel. Additional benefits imparted to the guide dog handler include friendship, companionship, increased social-function, and improved self-esteem and confidence (1–10). More has been published about guide dog usage in the scientific literature than about other types of service/assistance dogs. According to York and Whiteside (11) this work has leaned toward aspects of training, health and reproduction, and benefits to mobility and well-being, with less on the experience of owning a guide dog. Compatibility and the success or failure of the relationship between a person and their first guide dog was assessed by Lloyd et al. (12), and the complexities of successful and unsuccessful guide dog matching and partnerships were examined by Lloyd et al. (13) and Lloyd et al. (14). These, and other studies (11, 15) indicate that factors other than orientation and mobility, such as the dog's social behavior in and out of the home environment, need to be considered in the process of matching a dog to its new owner to promote a successful outcome. However, limited evidence exists that discusses how the handler might be affected when a guide dog retires, is returned to the guide dog training establishment (for whatever reason) or dies.

The end of the guide dog partnership can result in reduced mobility (1, 2) and may have profound psychosocial effects on the handler due to feelings of bereavement (6, 16–19), and loss of self-esteem (17, 20, 21). A seminal study by Nicholson et al. (22) examined distress arising from the end of a guide dog partnership and concluded that the emotions experienced by the handler at this time could be likened to feelings that follow the death of a pet, the loss of a close friend or relative or the loss of sight. These findings are supported by Kwong and Bartholomew (23) who explored individuals' relationships with an assistance dog and concluded that when confronted with the loss of their dog, people experienced intense grief consistent with the loss of a caregiving relationship. More recently, Uccheddu et al. (24) conducted a comprehensive analysis of grief responses in dog owners after the death of a pet dog and found that owners tended to humanize their pets and experienced a negative view of life after the death of their pet. The grief response to losing a dog, be it a pet or an assistance dog, is still an underestimated issue. Given the increasing number of service/assistance dogs being used across the world (25–27), this type of grief is of major concern for the welfare of the people who use them.

The present study^1^ builds on these findings by discussing how feelings at the end of the guide dog partnership affect the handlers' relationships with subsequent guide dogs, and indicates trends in the dataset concerning multiple dog use. The effects of being without a guide dog, after experiencing guide dog mobility, on quality of life will also be discussed. A better understanding of the effects of the end of the partnership will help inform the guide dog industry of how best to support their clients during this time and when transitioning to another dog. Findings may be applied to other service/assistance dog organizations and pet (companion) dog ownership as the impact of the separation is similar in some aspects (28).

## Method

### Participant Recruitment

Customarily, guide dogs are well-accepted in New Zealand and guide dogs are provided to a wide-range of applicants with varying visual conditions and mobility needs (17). In order to apply for a guide dog, the applicant should be eligible to receive services from Blind Low Vision NZ (formerly Blind Foundation/Royal New Zealand Foundation of the Blind) by being blind or markedly sight impaired. Blind Low Vision NZ Guide Dogs is a member school of the International Guide Dog Federation (IGDF), and as such is accredited to the highest international standards. The population of interest, as previously described in Lloyd et al. (1), was all people living in New Zealand who were, or had been, clients of Blind Low Vision NZ Guide Dogs since its establishment in 1973. At the time of participant recruitment, this was ~210 people. No exclusion criteria were applied. For reasons of privacy, a Blind Low Vision NZ staff member mailed the invitations to participate on behalf of the researcher (first author). The invitations consisted of an information document (supplied in the person's preferred format of Braille, audiotape, e-mail, or regular or large print), plus a consent form and a pre-paid, addressed envelope. Potential participants returned the signed consent form directly to the researcher, thus maximizing confidentiality and anonymity. Seventy two percent (*n* = 151) of the target group responded, from which 50 participants were randomly selected (i.e., around one quarter of the entire population of guide dog users in New Zealand at this time). Those not selected were notified and thanked.

### Participants

Fifty people from across New Zealand (as described above) who had used one or more guide dogs were interviewed by the researcher either by telephone (78%) or face-to-face (22%) regarding their experiences with guide dogs. The total sample (people and dogs) is described in Lloyd et al. (13) and Lloyd et al. (14). Of these 50 people, 36 had experienced the ending of at least one partnership. These 36 people constitute the participants for the present study that explored experiences associated with the end of the guide dog partnership and how they affected subsequent matches. Just over half the sample identified as female (20, 55.6%), and the majority (25, 69.4%) identified themselves ethnically as New Zealanders of European decent, 6 (16.6%) as Māori (the indigenous people of Aotearoa/New Zealand), and the remainder as “other.” They ranged in age from 28 to 80 years, with a mean age of 50.6 years (SD = 14.0). All were registered members of Blind Low Vision NZ, with an affiliation from 4 to 66 years, and an average membership of 29.1 years (SD = 15.9). These characteristics were in accordance with Blind Low Vision NZ's estimation of its client base at the time of the study. At the time of the study 27 of the 36 participants were currently using a dog. Nine were not, and of these, seven had decided not to use a dog in the future due to not wanting a dog currently (*n* = 3) or at all (*n* = 3), or due to having a poor relationship with the guide dog school (*n* = 1). The other two were on the waiting list for a replacement dog. Nearly all participants (32, 88.9%) had used more than one dog, the average being 2.9. Consequently there were more handler-dog partnerships than there were participants in the study. Of the 36 participants; 17 had experienced a single partnership end (dog loss), 11 had experienced two losses, three had experienced three, one had experienced four, five and six losses, respectively, and two people had experienced the ending of seven partnerships. This makes a total of 77 ends of partnerships that were rated by the 36 participants.

### Data Collection

All 36 participants had encountered the ending of at least one partnership with a guide dog. Participants were asked to rate their experiences in terms of (a) what became of the dog (the dog's fate), (b) their feelings when the partnership ended, (c) how this affected their application for a subsequent dog, and (d) how the end of the partnership influenced the relationship with subsequent dogs. Issues concerning how being without a guide dog, after having experienced using one, affected quality of life were also explored. Participants were asked to describe, in order of importance, how the absence of the dog affected their quality of life in general. All questions were open ended. Thus, the participants provided unique, unanticipated answers, which were recorded in written form and analyzed descriptively to show how often a response was given *via* measures of frequency, including count and percent.

## Results: the End of the Partnership

### Fate of the Dog

Participants (*N* = 36) had experienced the ending of at least one and up to seven partnerships with dogs, giving a total of 77 “losses” (Table 1). Of these 77, 13 dogs (16.9%) were kept as pets by their handlers^2^ and another 24 were retired to live with a friend (10, 13.0%), family member (2, 2.6%) or rehomed by the guide dog school (12, 15.6%). Twenty-three (29.9%) of the dogs were returned to the guide dog school; Of these, 19 were rematched with another handler, three were withdrawn from the guide dog program and rehomed by the guide dog school, and one had a successful “change of career” with a different national working dog program. The remaining 17 (22.1%) dogs died of old age, underwent euthanasia for health problems or had a fatal accident before reaching retirement. Following the end of the majority of the partnerships (57, 74.0%) the handlers either wanted to or did keep in touch with their dogs. It was notable that people were most likely to want to retain contact with their first dog.

**Table 1:** Issues regarding the end of the handler-dog partnership (*n* = 77) for the handlers' first (1st dog) and subsequent guide dogs (up to the 7th dog used).

**Issues**	**1st dog** ** (*n* = 36)**	**2nd dog** ** (*n* = 19)**	**3rd dog** ** (*n* = 8)**	**4th dog** ** (*n* = 5)**	**5th dog** ** (*n* = 4)**	**6th dog** ** (*n* = 3)**	**7th dog** ** (*n* = 2)**
**Dogs' fate** ***n*** **(%)**							
Retired-family	2 (5.6)	0 (0.0)	0 (0.0)	0 (0.0)	0 (0.0)	0 (0.0)	0 (0.0)
Retired-friend	7 (19.4)	1 (5.3)	1 (12.5)	0 (0.0)	0 (0.0)	1 (33.3)	0 (0.0)
Retired-RNZFB home	7 (19.4)	3 (15.8)	1 (12.5)	1 (20.0)	0 (0.0)	0 (0.0)	0 (0.0)
Returned-rematched^*^	2 (5.6)	7 (36.8)	2 (25.0)	2 (40.0)	3 (75.0)	2 (66.7)	1 (50.0)
Kept as pet by handler	6 (16.7)	3 (15.8)	2 (25.0)	1 (20.0)	1 (25.0)	0 (0.0)	0 (0.0)
Returned-RNZFB's home	2 (5.6)	0 (0.0)	1 (12.5)	0 (0.0)	0 (0.0)	0 (0.0)	0 (0.0)
Returned-new career	0 (0.0)	1 (5.3)	0 (0.0)	0 (0.0)	0 (0.0)	0 (0.0)	0 (0.0)
Deceased/euthanasia	10 (27.8)	4 (21.1)	1 (12.5)	1 (20.0)	0 (0.0)	0 (0.0)	1 (50.0)
Kept in touch-if possible (%)	32 (88.9)	12 (63.2)	6 (75.0)	3 (60.0)	2 (50.0)	1 (33.3)	1 (50.0)
**Time “put off” applying for a replacement dog (%)**							
No time	28 (77.8)	15 (78.9)	7 (87.5)	5 (100.0)	4 (100.0)	2 (66.6)	1 (50.0)
Up to 3 months	3 (8.3)	1 (5.3)	0 (0.0)	0 (0.0)	0 (0.0)	0 (0.0)	0 (0.0)
3–6 months	1 (2.8)	0 (0.0)	0 (0.0)	0 (0.0)	0 (0.0)	0 (0.0)	0 (0.0)
6 months−1 year	0 (0.0)	1 (5.3)	0 (0.0)	0 (0.0)	0 (0.0)	0 (0.0)	1 (50.0)
2–5 years	2 (5.6)	0 (0.0)	0 (0.0)	0 (0.0)	0 (0.0)	0 (0.0)	0 (0.0)
Indefinitely	2 (5.6)	2 (10.5)	1 (12.5)	0 (0.0)	0 (0.0)	1 (33.3)	0 (0.0)

*No missing responses*.

* *Rematched dogs are dogs that are returned to the guide dog school and matched to another handler*.

### Application for a Replacement Dog

Overall, the end of a partnership did not put handlers off applying for another dog immediately in 62 (80.5%) of the 77 cases (Table 1). However, on six (7.8%) occasions people were put off indefinitely and nine (11.7%) chose to wait from a couple of months up to 5 years. Most of the people who had experienced a mismatch and who had applied for a replacement dog stated that they were optimistic about getting a better dog next time.

The people who wanted a replacement dog immediately had wanted guide dog assisted mobility as soon as possible and/or had kept their previous dogs as pets. For most this was the right decision, even if they had experienced an unsuitable dog, but some regretted not taking more time to come to terms with the loss of their previous dog before acquiring its replacement. On six occasions people indicated they would never get another dog and this was because they did not expect to get over the loss of their previous dog and/or they did not wish to repeat the painful experience of receiving an unsuitable dog. One who had initially felt this way declared that the guide dog school had “forced” another dog on her, which she was ultimately grateful for as it turned out to be a very good match. When people elected to wait some time before requesting another dog they did so as they needed time to grieve, wanted a break from the responsibility of owning a dog or had temporary changes in personal circumstances such as increased social support or an alternative means of travel.

Of the 36 participants, 26 predicted using another guide dog at some point, seven people did not, and three people said they would consider it. Of the 26 wanting to use another dog, eight did so solely because they preferred a dog to a long cane as a mobility aid, while 16 people, who also preferred the dog to the long cane, gave equal importance to the companionship and social interactions that the dog provided. Reasons given by the seven people not envisaging the use of a dog in the future included: changed mobility needs (*n* = 2); lack of trust in the guide dog school (*n* = 2); considered it not worth the effort of retraining with a new dog (*n* = 1); felt there was no difference in life with and without a guide dog (*n* = 1); or was in an unsuitable living environment (*n* = 1). The three participants who were undecided were enjoying a break from dog ownership (*n* = 1), unsure about future need (*n* = 1) or under pressure from family not to get another dog (*n* = 1).

Six participants (16.7%) were currently on the guide dog school's waiting list for a replacement dog; three currently had no dog, one had a temporary dog, another a poorly matched dog and the remaining person had a dog that was due to retire. The other 30 participants not currently on the waiting list comprised 19 people who were happily using their current dogs, three who were debating returning their current dogs (mainly for canine reasons of distraction, aggression and poor health), and eight who had previously used a dog and decided not to use another, or were having a break, as described earlier.

### Feelings at the End of the Partnership

Amongst the 36 participants, the end of 77 guide dog/handler partnerships (losses) had been experienced. Table 2 presents the responses to the question of how people felt at the end of a partnership with a guide dog with responses to the loss of the first (column 2) and subsequent dogs (columns 3-8) provided separately. The combined (total) responses are presented in column 9. From the total column it can be seen that the main feelings experienced at the end of partnerships were acceptance, extreme grief and feeling reassured about the dog's future home. There was a high degree of acceptance expressed, because people had enjoyed a successful relationship, and/or understood the rationale for an early ending to the partnership and/or needed another dog for mobility. Extreme grief was expressed at the end of around half of the relationships and was likened to losing a family member or other loved one. Grieving was not limited to the handlers; many of those expressing profound grief said that their family members and some work colleagues who had spent a good deal of time with the dog also suffered a great loss. Feelings of extreme grief were expressed at the end of 38 partnerships and the fate of these 38 dogs was: rehoming (*n* = 15); death during working life (*n* = 15); being rematched to another handler by the guide dog school (*n* = 5); and remaining with their handlers as pets (*n* = 3). While feelings of extreme grief were very common, experiencing a lesser degree of grief or feeling neutral was rare. Relief was expressed at the end of several partnerships (16.9%)—all of which were considered to be poor matches. It is notable that feelings of extreme grief were expressed most often after the loss of the first dog and feelings of relief that the partnership had ended were lower for first dogs compared to subsequent dogs combined.

**Table 2:** Feelings expressed about the end of the handler-dog partnership according to number of losses experienced (1–7).

Feelings^*^n (%)	**1st dog (*n* = 36)**	**2nd dog (*n* = 19)**	**3rd dog (*n* = 8)**	**4th dog (*n* = 5)**	**5th dog (*n* = 4)**	**6th dog (*n* = 3)**	**7th dog (*n* = 2)**	**Total!!break (*n* = 77)**
Grief-extreme	22 (61.1)	8 (42.1)	3 (37.5)	1 (20.0)	1 (25.0)	1 (33.3)	2 (100.0)	38 (49.4)
Grief-somewhat	3 (8.3)	1 (5.3)	0 (0.0)	0 (0.0)	0 (0.0)	0 (0.0)	0 (0.0)	4 (5.2)
Neutral	1 (2.8)	1 (5.3)	0 (0.0)	0 (0.0)	0 (0.0)	0 (0.0)	0 (0.0)	2 (2.6)
Relieved	1 (2.8)	4 (21.1)	1 (12.5)	3 (60.0)	2 (50.0)	2 (66.6)	0 (0.0)	13 (16.9)
Angry with the guide dog school	8 (22.2)	6 (31.6)	1 (12.5)	1 (20.0)	2 (50.0)	1 (33.3)	0 (0.0)	19 (24.7)
Shocked to “fail”	3 (8.3)	4 (21.1)	0 (0.0)	2 (40.0)	1 (25.0)	1 (33.3)	0 (0.0)	11 (14.3)
Guilty	11 (30.6)	2 (10.5)	0 (0.0)	0 (0.0)	0 (0.0)	0 (0.0)	1 (50.0)	14 (18.2)
Accepting	23 (63.9)	12 (63.2)	7 (87.5)	2 (40.0)	2 (50.0)	0 (0.0)	0 (0.0)	46 (59.7)
Reassured re. Pet home	16 (44.4)	6 (31.6)	7 (87.5)	2 (40.0)	2 (50.0)	0 (0.0)	0 (0.0)	33 (42.9)
Worry re. mobility	3 (8.3)	2 (10.5)	1 (12.5)	0 (0.0)	1 (25.0)	0 (0.0)	1 (50.0)	8 (10.4)
Resentful/denial	2 (5.6)	1 (5.3)	2 (25.0)	0 (0.0)	0 (0.0)	0 (0.0)	0 (0.0)	5 (6.5)
No self-blame	1 (2.8)	2 (10.5)	0 (0.0)	0 (0.0)	0 (0.0)	0 (0.0)	0 (0.0)	3 (3.9)
Family devastated	6 (16.7)	3 (15.8)	1 (12.5)	1 (20.0)	0 (0.0)	1 (33.3)	1 (50.0)	13 (16.9)
Hoping for better next time	2 (5.6)	0 (0.0)	0 (0.0)	1 (20.0)	0 (0.0)	0 (0.0)	0 (0.0)	3 (3.9)

* *Total percent adds to more than 100 due to open-ended questions/multiple responses*.

Acknowledging that the working relationship was over was easier for the majority of participants if they knew the dog was going to a good home. This was especially true if the dog was being replaced because of work, or was being kept as a pet. A few people found the situation very hard to accept, and resented and in some cases denied that the dog was getting too old to work. Loss of mobility was a concern at the end of eight partnerships as people worried about losing their freedom and independence.

Anger directed toward the guide dog school was experienced at the end of 19 partnerships because people felt that they had not been supplied with a suitable dog in the first place and/or were not fully informed that a dog had a problematic history. Other reasons for ire at this time came from people who felt abandoned when their partnerships ended because of the dogs' ill health, due to a perceived lack of emotional support from the guide dog school. Guilt was experienced at the end of 14 partnerships, 11 of which were first dogs, not only due to having to give up the “old” for the “new,” but in some cases where dogs had died people felt guilty that the dog had not been able to enjoy retirement and when a partnership had failed, some people felt guilty that it may have been their (or their family's) fault. “Failure” had not been an option considered by the 11 people who were shocked when their relationship did not work out necessitating in the return of the dog.

### Relationships With Subsequent Dogs

Not everyone continued on to use another guide dog when a partnership was over so responses to the question of how a relationship with a previous dog had influenced the next was relevant following the end of 71 partnerships. Of these, 19 (26.8%) were reported to have had a negative effect in that the old dog was considered to be a better mobility aid and/or less puppy-like in general, and/or that the memory of the old dog inhibited bonding with the new. The latter was reason enough to put a few handlers off acquiring a replacement dog indefinitely. There were 13 (18.3%) examples of a positive effect through the handler knowing what to expect through experience and realizing that the new dog was an improvement over the previous one. No comparison was made following the end of 12 (16.9%) partnerships as the dogs were appreciated as individuals despite the associated feelings of loss. However, the largest number 27 (38.0%) were said to have had no effect on the subsequent relationship. Breed-specific behaviors also played a role, as exemplified by two people saying they did not want to repeat the experience of having a Labrador retriever due to the scavenging behaviors exhibited.

### Post-guide Dog Assisted Mobility: Effects on Quality of Life

Responses to the question of how quality of life was affected by being without a guide dog after experiencing guide dog mobility are presented in Table 3. Most people provided more than one response so the number of comments (89) was greater than the number of participants (*N* = 36) and percentages add to more than 100. Seventy-six of the 89 comments (85.4%) indicated that quality of life was reduced when participants were without a dog after experiencing guide dog mobility. This outcome was mainly due to a reduction in independent mobility for 31 (86.1%) participants, followed by losing a friend and companion for 21 (58.3%). Other reductions in quality of life included the effect on social interactions for 10 (27.7%) and loss of self-esteem and/or confidence for six (16.7%). Quality of life had increased for the three (8.3%) who enjoyed a break from the responsibilities of looking after a dog, the two (5.6%) who appreciated not having to deal with an unsuitable dog and the one (2.8%) whose long cane skills improved through the opportunity to practice. The remaining seven comments indicated that quality of life did not change with four people (11.1%) explaining that their requirement for a dog had altered as a result of a change in circumstances re their mobility needs, and three (8.3%) said that they were good cane travelers and had not felt particularly emotionally attached to the dog. In responding to this question participants were asked to describe the most important effect first and, as shown by the asterisks in Table 3, the results mirror the overall findings. Not only was loss of individual mobility the most commonly cited effect of no longer having a canine guide, it was also considered to be the most important effect on quality of life. This was followed by loss of friend or companion being the second most important effect and the curtailment of social interactions the third most important and frequent.

**Table 3:** The participants' (*N* = 36) comments on how being without a guide dog after experiencing guide dog assisted mobility affected quality of life.

**Effect on quality of life**	**Comments**	***N* (%)**
**Reduced**	Loss of independent mobility^*^	31 (86.1)
	Loss of friend/companion^**^	21 (58.3)
	Curtailing of social function/interactions^***^	10 (27.7)
	Loss of self-esteem/confidence	6 (16.7)
	Concern regarding when dog will be replaced	5 (13.9)
	Feelings of failure re mismatch/guilt at giving up dog	3 (8.3)
**Neutral**	Handler's decision—change in circumstances/mobility needs	4 (11.1)
	No effect—good cane traveler and not very attached to dog	3 (8.3)
**Increased**	Appreciated a break from responsibilities of dog ownership	3 (8.3)
	Return of unsuitable dog liberating	2 (5.6)
	Long cane skills improved	1 (2.8)

*Total percent does not add to 100 due to open-ended questions/multiple responses*.

* *Most important effect*;

** *Second most important effect*;

*** *Third most important effect*.

## Discussion

One way of understanding humans' relationships with companion animals is through attachment theory i.e., the concept that we become emotionally attached to our companion animals in a similar way as we do to people. The human-animal bond has existed for thousands of years. The American Veterinary Medical Association (AVMA) defines this bond as “a mutually beneficial and dynamic relationship between people and animals that is influenced by behaviors essential to the health and well-being of both. This includes, among other things, emotional, psychological, and physical interactions of people, animals, and the environment.” (42). Dogs (and other animals) have been helping people with physical disabilities and providing emotional support for centuries (25, 29). The results of the present study shows that the existence of this bond has major significance for the well-being of both the handler and the dog as it influences how people feel about getting a subsequent guide dog and the relationship formed between the dyad, as well as directly impacting on the handlers' well-being at the end of the partnership.

### Distress at the End of the Partnership

Almost all the participants had used more than one dog. As the transition from one dog to the next is a recurring feature, guide dog handlers probably experience the end of more relationships than the average pet dog owner (22). Retiring a guide dog is not only difficult for the handler, but also for the handler's family and friends, and doubtlessly the dog. Most people expressed feelings of grief when a working partnership ended, whether it was successful or not. Extreme grief was the feeling most frequently reported and this was shared by family members, friends and co-workers. The depth of emotion was compared to losing a family member or other loved one; a comparison that was also reported by Fogle (30) and Stewart (31) at the end of some person and pet relationships, and more recently by Uccheddu et al. (24). The distress caused by the end of the partnership between a handler and a guide dog might be more intense than that experienced between a person and a pet (8) due to the interdependent nature of the relationship (7, 10), the time spent together and because the dog helps the handler to do things that could not be accomplished alone. However, the grieving process over the loss of any companion animal may be hampered by the lack of validation for the mourning of non-human animals by the general public and some professionals, as well as the lack of socially sanctioned grief rituals that typically accompany the loss of a human (32). Many guide dog schools around the world recognize this significance and have memorial gardens for people to visit, remember and honor their guide dogs who have passed.

A study by Barnard-Nguyen et al. (33) that measured people's responses to the loss of a pet (dog or cat), rather than a human, found that people had different types of grief (sorrow, anger, and guilt). While a reaction of sorrow on the loss of the pet was considered to be part of a “normal” psychological process, some people developed “complicated” grief manifesting as depression and other mental health problems. From the attachment theory perspective, it would be expected that people with a stronger attachment to their guide dog or pet would feel more grief when the pet dies, which was the case in both the present study and Barnard-Nguyen et al. (33) study. Barnard-Nguyen et al. (33) found people who were more emotionally attached to their pet reported more grief and sorrow, and also more feelings of anger (e.g., toward the veterinarian for not being able to save the pet), but not guilt. This contrasts somewhat with the end of the working relationship with a guide dog as described in the next paragraph.

The participants that described the end of the partnership with their guide dog as a relief were commenting on dogs that they felt they had been mismatched with, suggesting a strong emotional bond had not been formed. This finding was also discussed in a focus group prior to the present study (34). However, some participants in the present study reported anger at being mismatched, shock in having “failed,” guilt in case it had been their fault, and a few lost self-esteem and confidence. This was true even if the team had not bonded. These findings parallel those of Nicholson et al. (22) who found that the end of the partnership was an upsetting experience, even if there had been problems in the relationship. The exception to this was mismatches that ended after a relatively short period with no real bonding.

A related study by Lloyd et al. (13) and Lloyd et al. (14) on the complexities of successful and unsuccessful guide dog partnerships found that most dogs that were considered to be mismatched were returned to the guide dog school by the handler after just 3 months. An earlier study by the same authors (34) reported that the bond between a handler and their new guide dog could take 6 months or longer to develop. Therefore, guide dog instructors should inform handlers who may be frustrated with a new partnership that the relationship might take from 6 months up to a year to improve. Lloyd et al. (13) and Lloyd et al. (14) also found that some handlers who returned their problem dog did not feel that a mis-match had occurred because the guide dog instructor discussed potential issues at the outset, thus enabling the handlers to make informed choices. Hence, the opportunity for person and dog to work through problems together may actually strengthen the bond.

The grieving process was easier if handlers were reassured about the dogs' destiny, an outcome also reported by Nicholson et al. (22). This held true whether dogs were being kept as pets or were going to approved retirement homes. As Sanders (6) illustrated, the latter option was seen as a better alternative for dogs in that they were not expected to cope with the presence and/or the role of a new guide dog. It is noteworthy that almost all who experienced the death of a dog in the present study while it was still working experienced feelings of extreme grief at the end of the partnership. This may not only be due to attachment dynamics but may also be due to the loss of the equally important caregiving relationship as described by Kwong and Bartholomew (23). According to Bowlby (35), the care giving system is designed to provide protection and support to a dependent—goals that are no longer viable on the death of a dog and which may lead to feelings of intense despair. Identifying those who may be at greater risk for problematic grief reactions is of considerable value to guide dog schools. While guide dog schools should be prepared to support all clients in their grief responses, recognizing that someone is highly attached to their dog or that the dog died during its working life should trigger additional support.

### Applying for a Replacement Dog

As for those who mourn the end of a relationship with humans, feelings of grief for the end of the relationship with a service dog or pet dog should be acknowledged and respected. The need for a period of adjustment before committing to a relationship with a new service dog or pet vs. prompt replacement is controversial. A study by Jarolmen (36) compared grief and bereavement responses of children, adolescents and adults who had lost a pet within a 12-month interval. Her findings indicated that children and adolescents are similarly attached to their pets and that children grieved more than adults did. Jarolmen (36) also concluded that the more recent the loss the more intense the response, but if the loss is anticipated grief is allayed. In her doctoral thesis abstract, Jarolmen (37) makes the interesting statement that those who have another pet in the home at the time of loss grieve the same for the lost pet as those who do not have another pet in the home, but those who replace the pet have a higher grief response. Unfortunately, it was not possible to obtain a copy of the entire thesis to read more about this relationship. Stewart's (31) findings suggested that deferring replacement was not warranted, even when a highly significant pet died, provided the pet's death was not trivialized and the new pet was introduced in a sensitive manner.

The majority of handlers in the present study said that they would continue to use guide dogs in the future, as they preferred and/or had become dependent on guide dog assisted mobility. Those who did not anticipate using another dog had a limited workload, unsuitable living conditions, family pressure, or did not have a trusting relationship with the guide dog school. Others, who were undecided, enjoyed not having the responsibility of ownership, were concerned that they might experience another mismatch or were impartial concerning cane or dog. Although the grief response was high, this did not preclude most handlers from applying for another dog right away, including those who had experienced a mismatch. The decision about when to replace a dog is personal, but some handlers regretted replacing their dog before adjusting to the loss of the previous one. Regardless, it appears that the need for mobility is the force behind the desire to replace a dog quickly.

Many handlers felt that the end of the partnership with the previous dog had a negative effect on the relationship with the new one. This was due to the old dog being considered a better mobility aid, less puppy-like and the memory of the previous dog inhibiting bonding. The role of the guide dog instructor re the human-animal bond is vital at this time to maximize the potentials of the relationship between their clients and their new dogs. Practical implications for the guide dog industry include that feelings of grief at the end of a partnership should not be trivialized and the new dog should be introduced in a sympathetic manner. Guide dog schools that offer grief-counseling sessions to enable handlers to share their feelings over the loss of the previous dog, have found that these handlers form a healthy relationship and train more quickly with the replacement dog (J. Campbell, Leader Dogs for the Blind, Michigan, USA, personal communication, August, 2000). Thus, the grief response and its expression may in fact be necessary in order for the next bond to form.

### Quality of Life

Lloyd (17) showed that using a guide dog improved the quality of life for handlers due to enhanced mobility, social interactions, fitness, mental and physical health, and adjustment to loss of vision. These findings were echoed by a recent longitudinal study by McIver et al. (38) who demonstrated that guide dog owners perceived their quality of life to increase over time in similar terms. However, as shown by the present study, quality of life can also decrease at the end of some partnerships. Lessening of quality of life in the present study was due to a reduction in independent mobility followed by the loss of a friend and companion and curtailment of social interactions. People experience a reduction in the quality of their independent mobility for a variety of reasons. For example, Lloyd et al. (2) found that it was troublesome for those who were accustomed to guide dog assisted mobility to be without a dog because their cane skills had deteriorated. The present study also reveals that confidence and self-esteem were reduced when an unsuitable dog was received, or when a dog was retired or died. These effects were also found in children (39) and adults (32, 40) who mourned the loss of a pet, and are described for guide dog owners in Schneider (21) and Gosling (20).

It would behoove guide dog schools to be empathic to the emotional and practical challenges their clients face at this time. Many guide dog schools provide access to councilors or information sources that may be helpful to people dealing with the loss of a guide dog such as helplines, books, on-line resources etc. Since this research was conducted Blind Low Vision NZ set in motion a client-driven national system for grief management, including sharing memories/experiences, for members who have lost or retired a guide dog, and Ward and Pierce (19) created a client driven information resource for second time guide dog applicants to help them transition to a new dog.

Practical implications for the guide dog industry can also be found in a practice report written by an experienced guide dog handler (21). Schneider suggests: being honest, letting the person know that one is open to hearing about one's grief, and reminding others that all dogs were once new and young. Allen (16) has written a heart wrenching account entitled “Letting go of the harness for the last time” that provides advice for guide dog schools and for veterinarians who care for guide dogs (and thus the person-dog team). Allen (16) study illustrates how peoples' experiences can influence broader social issues including policies for agencies that provide guide dogs, and the role of the veterinarian when it is time to make decisions about retiring or euthanizing the dog.

### First vs. Subsequent Dogs

“Second dog syndrome” (SDS) is a phenomenon seen whereby a person's second guide dog is significantly less favored than the first dog (17) and is apparent on a number of levels in the present study. Lloyd (17) found that handlers' expectations regarding mobility were met less often and handlers were less compatible with their second dogs than their first dogs, with little apparent difference between the first and the third dog. Lloyd et al. (13) Lloyd et al. (14) showed that the number of dogs that were deemed to be mismatched and/or returned to the guide dog school were highest for second dogs compared to first or third. Similar trends were apparent in the present study regarding the handlers' feelings at the end of the partnership with their first dogs compared to subsequent dogs, where people grieved more; reported more feelings of guilt and less of relief when the partnership ended; and more desire to keep in touch with their first dogs. As explained in Lloyd et al. (13) and Lloyd et al. (14), it is understandable why there should be a “first-dog” effect in the handlers' affections; this dog initialized/improved independent mobility, thus being the catalyst for life changing events. Another explanation proposed by Lloyd et al. (18) is “distortion of memory” where handlers may have forgotten that their first dog was as boisterous and exuberant in its youth as the new dog now was, and that it took time for the dog (and the partnership) to mature. In addition, handlers may forget or deny that the previous dog made the same mistakes as the inexperienced new one, or harbor the unrealistic expectation that the new dog can take over precisely where the old dog left off (41). This is exemplified in a touching and elegantly written account by a graduate of Leader Dogs for the Blind on her experiences concerning the loss of several guide dogs:

Jack was my second dog. As I was training with him, we waited for the [traffic] light to change… I stepped from the curb with confidence. Jack executed a perfect (sic) diagonal crossing… [(41), pp.10-11].

Although this experience left the handler “dazed and frightened at the (wrong) corner,” this “green” dog eventually grew into a conscientious worker and Smith (41) emphasizes the importance of patience and understanding within any new relationship. Allen [(16), p.9] suggests that SDS can happen with any dog as “left overs from a previous dog can get in the way of accepting a new dog.” Knowing that a second, or any subsequent, dog is likely to be considered second best is valuable knowledge for guide dog instructors to help prepare clients to receive their next dog. Knowing that SDS is a tangible occurrence may alleviate some negative feelings the replacement dog engenders simply by not being the other dog.

## Conclusions

This study has contributed to the small body of literature concerning how the end of the partnership with a guide dog affects the handlers' quality of life and their relationships with subsequent dogs. Results support the negative view of life after the loss of a guide dog (16, 22) or other assistance dog (24), and mirrors many aspects of grief responses in pet dog ownership (28, 30, 31, 33, 36)—issues that remain underestimated. Although the grief response was high, this did not preclude most handlers from applying for a replacement dog quickly as the need for mobility was high. Future research could attempt to further tease apart any differences in a guide dog handler's reactions to the loss of their subsequent dogs—an exercise that was not feasible with the small number of successive dogs used in this study. It would also be beneficial to look at the experiences of handlers at various stages of working with dogs vs. having a break from guide dog use. Concerning how individuals handle the end of the partnership with their guide (or other) dogs: in the words of Gosling [(20), p. 12] “there is no right or wrong; it just is.” However, findings from the present study will help inform the guide dog industry regarding how best to support their clients during this time and when transitioning to another dog. Understanding the importance of the human-animal bond and implementing strategies to help clients grieve to support quality of life when experiencing the loss of a guide dog may be applied to other assistance/service dog users and the pet owning community.

## Data Availability Statement

The datasets for this article are not publicly or otherwise available due to issues of confidentiality. However, Supplementary Material can be found online at https://www.frontiersin.org/article/10.3389/fvets.2016.00114/full#supplementary-material.

## Ethics Statement

The studies involving human participants were reviewed and approved by Massey University Human Ethics Committee. The patients/participants provided their written informed consent to participate in this study.

## Author Contributions

JL undertook the research and wrote the article with CB with the approval of the other authors who have critically revised the content. All authors on this publication have contributed to the conception and the design of this work, and agree to be accountable for the content.

## Conflict of Interest

The authors declare that this study received funding from Douglas Pharmaceuticals Ltd. and the Palmerston North Medical Research Foundation. The funders were not involved in the study design, collection, analysis, interpretation of data, the writing of this article or the decision to submit it for publication.

## Footnotes

^1^Preliminary results of the present study were published in the proceedings of the 13th International Mobility Conference (18).

^2^According to some participants in this study, before 1988 the guide dog school regulations did not always permit handlers to apply for a replacement dog if they kept a retired guide dog as a pet, nor was contact between the former handler and the person/family that adopted a retired guide dog allowed.
